# A mathematical model of mechanotransduction reveals how mechanical memory regulates mesenchymal stem cell fate decisions

**DOI:** 10.1186/s12918-017-0429-x

**Published:** 2017-05-16

**Authors:** Tao Peng, Linan Liu, Adam L MacLean, Chi Wut Wong, Weian Zhao, Qing Nie

**Affiliations:** 1 Department of Mathematics, Center for Complex Biological Systems, and Center for Mathematical and Computational Biology, University of California, Irvine, CA 92697 USA; 2 Department of Pharmaceutical Sciences, Department of Biomedical Engineering, Department of Biological Chemistry, Sue and Bill Gross Stem Cell Research Center, Chao Family Comprehensive Cancer Center & Edwards Life sciences Center for Advanced Cardiovascular Technology, University of California, 845 Health Sciences Road, Irvine, CA 92697 USA

**Keywords:** Mesenchymal stem cell, ECM, *YAP/TAZ*, Cell fate decision, Stiffness sensing, Memory, Bistability, Nonlinear dynamics, Mathematical modeling

## Abstract

**Background:**

Mechanical and biophysical properties of the cellular microenvironment regulate cell fate decisions. Mesenchymal stem cell (MSC) fate is influenced by past mechanical dosing (memory), but the mechanisms underlying this process have not yet been well defined. We have yet to understand how memory affects specific cell fate decisions, such as the differentiation of MSCs into neurons, adipocytes, myocytes, and osteoblasts.

**Results:**

We study a minimal gene regulatory network permissive of multi-lineage MSC differentiation into four cell fates. We present a continuous model that is able to describe the cell fate transitions that occur during differentiation, and analyze its dynamics with tools from multistability, bifurcation, and cell fate landscape analysis, and via stochastic simulation. Whereas experimentally, memory has only been observed during osteogenic differentiation, this model predicts that memory regions can exist for each of the four MSC-derived cell lineages. We can predict the substrate stiffness ranges over which memory drives differentiation; these are directly testable in an experimental setting. Furthermore, we quantitatively predict how substrate stiffness and culture duration co-regulate the fate of a stem cell, and we find that the feedbacks from the differentiating MSC onto its substrate are critical to preserve mechanical memory. Strikingly, we show that re-seeding MSCs onto a sufficiently soft substrate increases the number of cell fates accessible.

**Conclusions:**

Control of MSC differentiation is crucial for the success of much-lauded regenerative therapies based on MSCs. We have predicted new memory regions that will directly impact this control, and have quantified the size of the memory region for osteoblasts, as well as the co-regulatory effects on cell fates of substrate stiffness and culture duration. Taken together, these results can be used to develop novel strategies to better control the fates of MSCs in vitro and following transplantation.

**Electronic supplementary material:**

The online version of this article (doi:10.1186/s12918-017-0429-x) contains supplementary material, which is available to authorized users.

## Background

Changes in cellular state can be regulated by mechanical signals from the cellular microenvironment, such as the local extracellular matrix (ECM) stiffness [1–4]. Recent studies into mechanotransduction have demonstrated that cells sense and integrate mechanical cues from the ECM, causing transcriptional changes to occur and influencing cell fate decisions [1–3, 5]. Mesenchymal stem cells (MSCs) are controlled by signals from the ECM and exhibit a wide range of differential gene expression patterns [1, 6]. The mechanisms governing how MSCs sense the surrounding ECM, and the myriad other factors affecting MSC fate, including interactions with proteins and ligands, tethering, and porosity, remain incompletely defined [3, 7]. Further understanding of how differentiation cues are mediated by mechanical stimuli will help to facilitate new biomaterial design, cell-based therapeutics, and engineered tissue constructs for use in regenerative medicine.

**Fig. 1:**
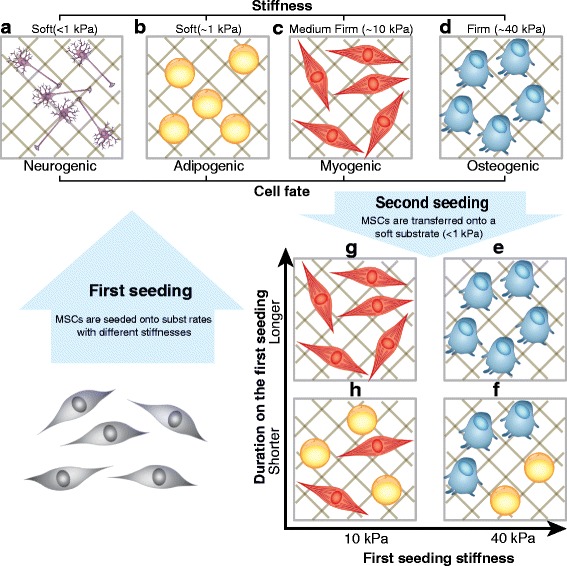
Mesenchymal stem cells (MSCs) exhibit mechanical memory. **a**, **b**, **c**, **d**: MSCs differentiate into distinct lineages under different substrate stiffness conditions by upregulating lineage marker genes TUBB3 (<1 kPa stiffness, the neurogenic fate), PPARG (~1 kPa stiffness, the adipogenic fate), MYOD1 (~10 kPa stiffness, the myogenic fate), or RUNX2 (~40 kPa stiffness, the osteogenic fate). When re-seeded onto a soft substrate (~1 kPa), MSCs are expected to undergo adipogenic differentiation [1, 6, 64]. **e**, **f**: However, for higher first seeding stiffness values (>10 kPa), or for long first seeding durations (>10 days), mechanical memory leads to heterogeneous osteogenic differentiation [8]. **g**, **h**: The model predicts that for high first seeding stiffness values (~10 kPa), or for long first seeding durations, mechanical memory leads to heterogeneous myogenic differentiation

The signals arising at the stem cell/substrate interface are complex and dynamic [7], however it has been shown that stiffness alone is enough to direct MSC differentiation [3, 4]. MSCs undergo neurogenic or adipogenic differentiation on soft substrates (<1 kPa), and myogenic or osteogenic differentiation on stiff substrates (>10 kPa) [1, 5] (Fig. 1). Upon further study, more complex differentiation patterns emerge. For example, it has been observed that cells cultured for a period of time on stiff substrates, such as standard tissue culture polystyrene (TCPS) plates, differentiate into osteogenic lineage cells even after being transferred from the stiff to a softer substrate [8]. Seeding MSCs on a phototunable substrate demonstrates that osteogenic patterns of gene expression persist even after decreasing the stiffness of the substrate [8]. This “mechanical memory”: the ability of MSCs to remember previous physical stimuli depends on both culture time and substrate stiffness (depicted in Fig. 1).

Due to mechanical memory, MSC differentiation in vitro can yield unpredictable (and undesirable) results. Mechanical memory also makes it very difficult to perform certain in vitro assays reliably, for example on extremely soft or stiff substrates, or assays with very long or short incubation periods. Such extreme culture conditions are nonetheless important to assess in order to fully elucidate the relationship between MSC fate and substrate stiffness [9]. In addition to the impracticality of performing short (i.e. seconds) or long (i.e. months) incubation experiments, experimental knock-downs of key genes involved in mechanotransduction, such as Yes-associated protein (*YAP*), can be lethal or highly toxic in vitro and in vivo [10, 11]. There is thus a need for *in silico* studies to simulate culture conditions and to map the MSC fate predictions to experimental results describing mechanically induced cell differentiation.

Several mathematical models of mechanotransduction have been built to describe cell differentiation directed by external mechanical stimuli [12, 13]. These include, for example, analysis of the role of *YAP/TAZ*, the transcriptional factors *YAP* and transcriptional co-activator with PDZ-binding motif (*TAZ*), in mechanosensing [14], and models that aim to predict cell differentiation during bone healing [12, 15, 16]. Mousavi et al. developed a 3D mechanosensing computational model to illustrate that matrix stiffness can regulate MSC fates. Their simulation results of MSC differentiation in response to substrate stiffness are in agreement with published experimental observations [13]. Burke et al. built a computational model to test whether substrate stiffness and oxygen tension regulate stem cell differentiation during fracture healing [12]. Their model predicted the presence of major processes involved with fracture healing, including cartilaginous bridging, endosteal and periosteal bony bridging, and bone remodeling, using parameters related to cell proliferation, oxygen tension, and substrate stiffness. However, these models are limited in that the effects of regulatory factors were not considered [12–16]. Furthermore, these studies used different models to represent different experimental observations. Hence it is difficult to describe the overall cell state space and to study the transitions between cell fates [12–16]. Thus, there is a need for a dynamic mathematical model, which can stimulate a continuous range of stiffness values and their associated cell fates.

**Fig. 2:**
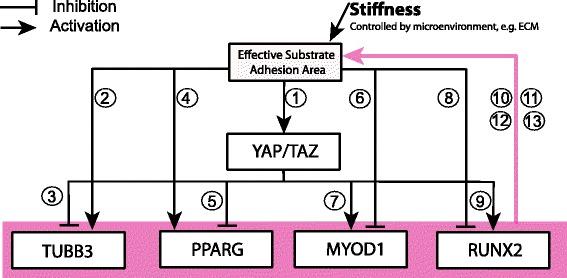
Regulatory network used to construct the mathematical model. The boxes represent genes or factors involved in MSC differentiation and the lines with *arrows* and with *bars* denote gene activation and inhibition respectively. External stiffness affects the substrate adhesion area. The *pink line* with an *arrow* denotes regulations by all species within the pink box. The circled indices refer to experimental evidence for each interaction, details of which are given in Table 1

Here we present a mathematical model of MSC differentiation controlled by the following set of core mechanisms (Fig. 2 and Table 1) [1, 6, 9]. The MSCs sense the stiffness of their environment directly via their adhesion to the substrate. The transcriptional factors *YAP* and *TAZ* mediate the signal via their interaction with downstream genes involved in cell differentiation. *TUBB3*, a gene encoding Tubulin beta-3 chain tightly correlated with a neurogenic cell fate is expressed when MSCs receive stimuli from a soft stiffness environment (<1 kPa) [1]. *PPARG*, peroxisome proliferator-activated receptor gamma, encodes an adipogenic marker and has been shown to be turned on in soft stiffness environments (~1 kPa) [6]. *MYOD1*, myogenic differentiation 1, a myogenic gene turned on in medium-stiff environments (~10 kPa), encodes key factors regulating muscle differentiation [1]. *RUNX2*, runt-related transcription factor 2, an osteogenic gene which is upregulated in high stiffness environments (~40 kPa), is a key transcriptional factor involved in osteoblast differentiation [1] (Fig. 1). We use this set of four lineage-specific genes in our model to minimally describe the transcriptional changes observed during MSC differentiation into four distinct cell fates under the influence of mechanical stimuli mediated by *YAP/TAZ* signaling.

**Table 1:** The references of regulatory interactions in the network

Index of Arrows	Interactions	References
1	*YAP/TAZ* is identified as mechanical sensors and mediators.	Halder, G et al, 2012; Dupont S. et al. 2011. [6, 18]
3	The inhibition of *TUBB3* can be attenuated by *YAP* depletion.	Alarcon, C et al. 2009 [65]
5	*PPARG* can be bound to *TAZ*, which results in transcription inhibitions from the aP2 promoter.	Hong, J.H. et al, 2006.[21]
7	*TAZ* functions as an enhancer of *MYOD*-mediated myogenic differentiation.	Jeong, H. et al, 2010. [66]
9	*RUNX2* has binding domain to *TAZ* for osteocalcin expression.	Hong, J.H. et al, 2006. Hong, J.H. et al, 2005 [20, 21]
10,11,12,13	Increased cell spreading results in higher stiffness sensitivity via increased binding of integrins to the ECM.	Halder G et al, 2012. Sun Y et al, 2012. Bernabe B P et al, 2016. [6, 17, 67]
2,4,6,8	These arrows are necessary for the dynamics of *TUBB3*, *PPARG*, *MYOD1*, and *RUNX2* on all possible stiffness environment since *TUBB3*, *PPARG*, *MYOD1*, and *RUNX2* are expressed only on the super soft stiffness (< 1 kPa), the soft stiffness (~1 kPa), the medium stiffness (~10 kPa), and the high stiffness (~40 kPa) environment respectively.	Engler, A.J. et al,2006; Halder G et al, 2012 [1, 6]

Based on the proposed regulatory network structure (Fig. 2), we simulate gene expression dynamics under different mechanical dosings. Each *in silico* experiment describes MSCs cultured in two passages: a first seeding and a second seeding. The substrate stiffness for the first seeding and the duration of the first seeding are particularly important in cell fate determination of MSCs. We also discover an important role for the second seeding stiffness through our simulation studies. Crucially, this two-seeding setup permits mechanical memory to be observed and studied. We assess when cell fates are determined not only by the current substrate stiffness but also by past exposure and find that a memory region exists for each of the four MSC-derived cell lineages studied. Our model demonstrates that stiffness-based MSC differentiation results from non-cooperative regulation of representative genes. Moreover, we show that lowering the second seeding stiffness of MSCs leads to a more diverse palette of MSC fates.

## Results

### A mathematical model based on a mechanotransduction network

The following set of biological assumptions has been used to develop the mathematical model. MSCs differentiate according to their surrounding mechanical environment [2–4, 6, 17]. Directed differentiation towards a particular lineage can be guided if the cells are cultured in a microenvironment that mimics the tissue elasticity of the environment in vivo [2, 3, 17]. Stiff substrates promote cell-ECM adhesion interactions via integrins [6]. These adhesive interactions control the localization of downstream transcriptional factors *YAP* and *TAZ*, which have been identified as mechanical sensors and mediators of such signals [6, 18]. *YAP/TAZ* localizes in the cytoplasm on soft substrates (~1 kPa) and can re-localize to the nucleus on stiff substrates (~40 kPa), thus functioning as a mechano-sensitive transcription factor [6, 18].

Additionally, *YAP/TAZ* has been reported to be an upstream factor of a number of genes associated with cell differentiation cues [6, 18, 19]. For example, the inhibition of *TUBB3* can be attenuated by *YAP* depletion, whereas that the factor *PPARG* binding to *TAZ* results in inhibition of transcription from the aP2 promoter [20, 21]. *TAZ* functions as an enhancer of *MYOD*-mediated myogenic differentiation. *RUNX2* can also bind to *TAZ* and cause osteocalcin to be expressed, thus promoting osteogenic differentiation [20, 21]. To describe these interactions, we model *YAP/TAZ* as both a downstream factor of the mechanical stimulus from the ECM and an upstream factor of the selected cell lineage genes [1, 22] (Fig. 2 and Table 1). Previous references show an intriguing relationship between morphological changes to MSCs and their lineage differentiation potential, whereby morphological changes have been shown to be instrumental to the process of MSC differentiation [1, 17, 18, 23–25]. In particular, it was shown that MSC osteogenic differentiation is enhanced by the morphological change of MSCs and *MYOD1* induced the myogenic differentiation efficiency via the morphological change of MSCs [26, 27]. Other factors regulating cell spreading such as *NKX2.5* were integrated in the model implicitly [28]. Therefore, we model a feedback loop between the lineage-specific target genes and the cellular sensing of substrate stiffness.

In order to predict how mechanical dosing influences MSC differentiation, we use ordinary differential equations to model the MSC lineage regulatory network [29–32] (Fig. 2 and Table 1). We assume that changes in the stiffness of the substrate act as stimulus to the cell (mediated by stiffness receptors) [12, 33]. We use Hill functions to model the chemical activation/inhibition [31, 32, 34]. We model the feedback loop that controls mechanical memory via a non-cooperative regulation, i.e., any of the lineage-specific genes (*TUBB3*, *PPARG*, *MYOD1*, *RUNX2*) can increase the effective stiffness adhesion area (we use “OR-GATE” logic). The feedback loop controls the expression of *YAP/TAZ* and its downstream genes via the stimulus (i.e., the change in stiffness [8]). We also test a feedback model of cooperative regulations (where *TUBB3*, *PPARG*, *MYOD1* and *RUNX2* must act together to increase the effective stiffness adhesion area, i.e. “AND-GATE” logic) but find that it does not satisfy the dynamical requirements of the MSC differentiation system (see Methods for full details).

### Model simulations predict mechanical memory regions for each lineage-specific gene

**Fig. 3:**
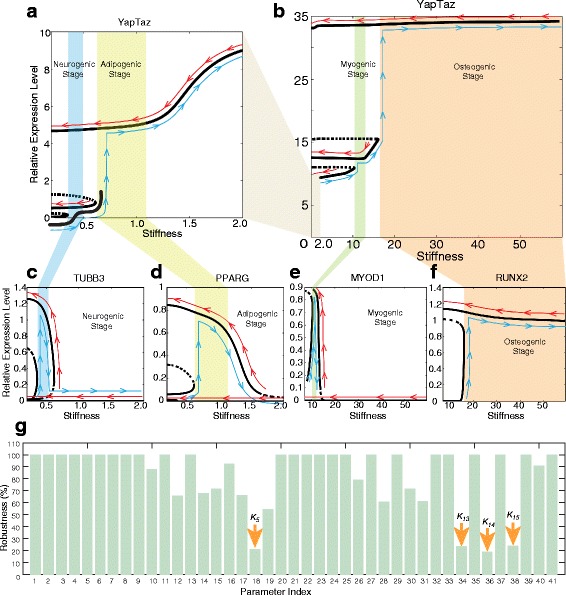
Multistability in the MSC differentiation network. The relative expression level of YAP/TAZ in a stiffness range from 0.1 kPa to 60 kPa is shown (**b**), with inset (**a**). The relative expression levels of lineage-specific genes are shown in (**c**-**f**). On each plot the x-axis is the stiffness of the substrate and the y-axis is the relative gene expression level. *Blue lines* illustrate changes in the relative expression level as the stiffness increases; *red lines* illustrate changes in the relative expression level as the stiffness decreases. (**g**). The robustness of the parameters in the mathematical model. The x-axis is the parameter index, corresponding to the notation of Table 2. The y-axis is the robustness of the parameters (defined in Methods)

The non-cooperative regulation model displays multiple steady states over the behavioral regions that we have investigated (with first seeding stiffness values ranging from 0.1 kPa to greater than 100 kPa; Fig. 3). This range is sufficient to encompass all known in vitro studies [1, 6, 8]. In Fig. 3a and b the multiple steady states of *YAP/TAZ* expression over the stiffness range studied are shown, and changes in the *YAP/TAZ* state can be visualized as the stiffness increases (blue lines) or decreases (red lines). The nonlinear relationship between *YAP/TAZ* and the stiffness of the substrate along the blue lines is consistent with previous observations [9, 19].

Figure 3c demonstrates bistability in the relative gene expression of *TUBB3* (driver of neurogenic differentiation) downstream of *YAP/TAZ. TUBB3* is “OFF” when the stiffness is lower than 0.2 kPa. It will be turned “ON” as the stiffness increases to 0.25 kPa. It turns “OFF” again as the stiffness increases further. Meanwhile, *TUBB3* stays “ON” when the stiffness decreases below 0.2 kPa, thus highlighting the mechanical memory observed during neurogenic differentiation. Notably, *TUBB3* stays “OFF” as the stiffness decreases from 0.6 kPa. We define the region of stiffness from 0.25 to 0.55 kPa as a “differentiation memory region” for *TUBB3*. This means that if the first seeding stiffness is within this range, the cell will “remember” the stiffness of this first seeding substrate, and will differentiate according (towards a neurogenic fate) upon re-seeding. Our model also predicts novel differentiation memory regions for *PPARG* (0.6 to 3 kPa; Fig. 3d) and *MYOD1* (10 to 15 kPa; Fig. 3e). *RUNX2* displays the largest differential memory region of the four lineage-specific marker genes studied.

Figure 3c-f collectively demonstrate a bistable region for each of the four lineage-specific genes studied. This is a startling prediction: that a region of mechanical memory exists for each of the cell fates, not just for osteogenic differentiation, as has been previously reported [8]. For neurogenic and adipogenic differentiation, the memory regions are smaller than that of osteoblasts yet may still be of great importance for stem cell fate regulation. The true contribution of each will require further study to elucidate, as a host of interacting factors contribute to the neurogenic and adipogenic cell fate decisions, including those which are not currently included in our model, such as the role of substrate-induced stemness and of epithelial to mesenchymal transition [35–37].

To test the robustness of the mathematical model we calculate the values of the robustness of each parameter in Eqs. (1,2,3,4,5 and 6) with respect to the memory and multistability of the system (full details of our methodology are in Methods). Out of the 41 parameters tested, 37 are robust to small changes for the majority of perturbations tested (and many of these 37 were robust more than 80% of the time) (Fig. 3g). Four parameters are found to be sensitive to small perturbations. All of these four parameters are involved in myogenic or osteogenic differentiation. Both these processes involve relatively large memory regions, thus it is possible that following these perturbations memory is maintained over parts of – but not the entire – original memory regions. Overall, we find that the system displays robustness using the parameters given in Table 2, with regard to the memory effects and the multistability of the states.

**Table 2:** Parameter values of the mathematical model

Index	Parameter	Value	Estimated from references	Index	Parameter	Value	Estimated from references
1	*k*_*1*_	0.2	[1,6]	2	*k*_*2*_	2.2	[1,6]
3	*k*_*3*_	5	[1,6]	4	*k*_*4*_	9	[1,6,8]
5	*k*_*5*_	4	[1,6]	6	*k*_*6*_	2.9	[1,6]
7	*k*_*7*_	3	[1,6]	8	*k*_*8*_	5	[1,6,8]
9	*k*_*9*_	2	[1,6,8]	10	*K*_*1*_	600	[1,6]
11	*n*_*1*_	4	[1,6]	12	*K*_*2*_	1.1	[1,6]
13	*n*_*2*_	2	[1,6]	14	*K*_*3*_	1300	[1,6]
15	*n*_*3*_	6	[1,6]	16	*K*_*4*_	0.8	[1,6,8]
17	*n*_*4*_	2	[1,6,8]	18	*K*_*5*_	20,000	[1,6]
19	*n*_*5*_	4	[1,6]	20	*K*_*6*_	1	[1,6]
21	*n*_*6*_	20	[1,6]	22	*K*_*7*_	60,000	[1,6]
23	*n*_*7*_	6	[1,6]	24	*K*_*8*_	1.1	[1,6]
25	*n*_*8*_	20	[1,6]	26	*K*_*9*_	0.1	[1,6]
27	*n*_*9*_	2	[1,6]	28	*K*_*10*_	0.5	[1,6]
29	*n*_*10*_	8	[1,6]	30	*K*_*11*_	0.89	[1,6]
31	*n*_*11*_	2	[1,6]	32	*K*_*12*_	4	[1,6]
33	*n*_*12*_	8	[1,6]	34	*K*_*13*_	12	[1,6]
35	*n*_*13*_	20	[1,6]	36	*K*_*14*_	3	[1,6]
37	*n*_*14*_	60	[1,6]	38	*K*_*15*_	16	[1,6,8]
39	*n*_*15*_	45	[1,6,8]	40	*K*_*16*_	4.5	[1,6,8]
41	*n*_*16*_	55	[1,6,8]	*d*_*i*_ (*i =* 1,2,⋯6)		1	Unconstrained

### A lower second seeding stiffness permits a greater number of MSC lineages

Potential energy landscape analysis is an appealing method with which we can investigate the system and study the MSC differentiation propensities under different conditions [38–40]. Since it is not possible to write down a complete expression for the potential energy of the system, we use an approximate method derived from mean field theory in order to calculate quasi-potential in terms of the six system variables [40, 41]. Explicitly, we calculate the potential of the system as *U*(*X*) = − ln(*P*_*ss*_(*X*)), where *P*_*ss*_(*X*) is the total probability of the state vector *X*, and *X* describes all the states of the system [40, 41].

**Fig. 4:**
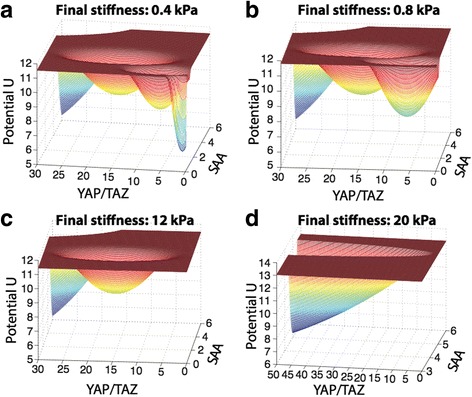
Potential landscapes of the regulatory network under different stiffness conditions. In each figure the relative stiffness level (input to the system) is plotted on the x-axis, the relative expression level of YAP/TAZ is plotted on the y-axis, the energy potential function U is plotted on the z-axis. Potential energy landscapes are shown with stiffness values of ~0.4 kPa (**a**), ~0.8 kPa (**b**), ~12 kPa (**c**) and ~20 kPa (**d**)

In order to visualize this potential function we project it onto a two-dimensional plane, defined by the species in our model: *YAP/TAZ*, and the effective stiffness adhesion area (SAA). In doing so we integrate out the four remaining system variables (*TUBB3*, *PPARG*, *MYOD1*, and *RUNX2*) [40, 41]. We are thus able to study how the potential depends on these variables for different stiffness values. In Fig. 4 we show the potential functions for four different conditions (we change the second-seeding stiffness values). Overall, we find that by reducing the second seeding stiffness, a greater number of steady states is permitted.

We simulate more than 10,000 initial conditions in order to avoid becoming trapped in local minima [40, 41]. We observe that across the entire landscape there are four stable states (or basins of attractions), representing neurogenic, adipogenic, myogenic, and osteogenic cell lineages. At a final stiffness of ~0.4 kPa, MSCs can differentiate into each of the four possible lineages (Fig. 4a). Only at such sufficiently small values for the second stiffness can MSCs differentiate into neurons: the basin of attraction for the neurogenic fate (i.e. the probability of differentiating into a neuron) is the smallest of the four fates. This means that mechanical memory is observed only over a small range of space. In comparison, a much greater mechanical memory effect is seen for the osteogenic lineage, corresponding to a larger basin of attraction. Figure 4b and c show the potential landscapes at second seeding stiffness values of ~0.8 kPa and ~12 kPa, respectively. The number of basins decreases to three, and then two, as the second seeding stiffness increases. When the second seeding stiffness increases further to ~20 kPa, we have only one remaining basin of attraction, thus only one possible cell fate: in this region the largest mechanical memory effect is seen, and osteogenic differentiation dominates. These data intriguingly suggest that simply by controlling the substrate stiffness upon re-seeding we can control the number of cell fates that are accessible to MSCs.

### The duration of the initial seeding determines the fate of an MSC

In addition to studying the effect of the second seeding stiffness on the fate of MSCs, we perform tests to assess the agreement between our model and in vitro observations regarding MSC differentiation [1, 18]. Specifically, we manipulate the stiffness of the second seeding substrate and the duration of the first seeding, and find, consistent with previous studies [5, 42], that both of these variables play an important role in the fate determination of an MSC upon differentiation. In addition these simulation results highlight several new phenomena.

**Fig. 5:**
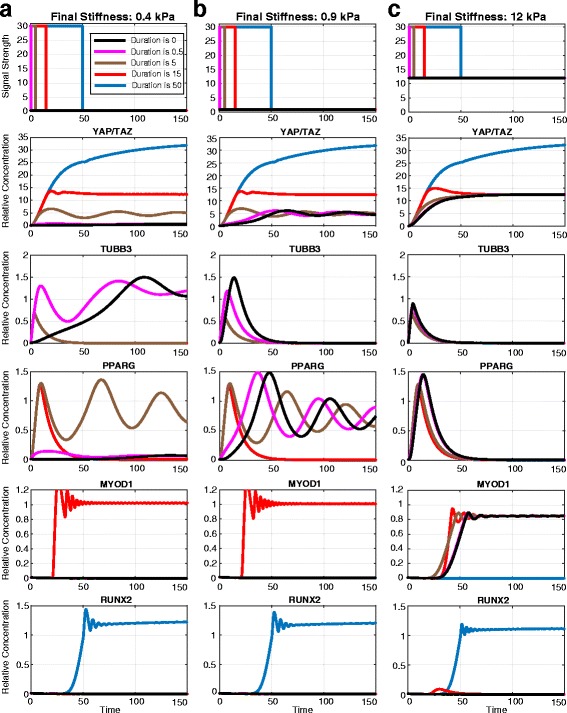
The duration of the first seeding regulates MSC fates via mechanical memory. The first seeding stiffness in this figure is 30 kPa. The second seeding stiffness is 0.4 kPa (**a**), 0.9 kPa (**b**) or 12 kPa (**c**). When the duration of the first seeding is 50 (*blue lines*), MSCs undergo osteogenic differentiation according to memory. When the duration of the first seeding is 15 (*red lines*), MSCs undergo myogenic differentiation. When the duration of the first seeding is 5 (*brown lines* in columns A and B), MSCs differentiate into adipocytes or myogenic cells. When the duration of the first seeding is 0.5 (*pink lines* in column A), MSCs are able to undergo adipogenic, myogenic, or neurogenic differentiation. Finally, when the duration of the first seeding is 0 (*black lines*), MSCs are able to undergo adipogenic, myogenic, or neurogenic differentiation

In order to examine how the first seeding duration affects MSC fates, we use a non-dimensionalized version of the model, that is, we express time in relative units. In Fig. 5a, the first and second seeding stiffness values are 30 kPa and 0.4 kPa, respectively. When the duration of the first seeding time is 50 (blue line), MSCs differentiate into osteoblasts (consistent with [5]): *RUNX2* is the only gene that is highly expressed under this condition. When the first seeding duration is 15 (red line), MSCs differentiate into skeletal muscle cells (*MYOD1* high); when the first seeding duration is five (brown line), MSCs differentiate into adipocytes (*PPARG* high). Finally when the first seeding duration is 0.5 or 0 (pink and black lines), MSCs differentiate into neurogenic cells (*TUBB3* high). These results are consistent with previous studies and highlight the breadth of control that mechanical memory enables: MSCs can be directed to four different fates by changing only the duration of the first seeding, keeping both of the first and the second substrate stiffness values constant. Although mechanical memory is not observed when the first seeding duration is less than 0.5, for the first seeding durations greater than five, we predict that mechanical memory will influence MSC fates, directing MSCs towards myogenic or adipogenic lineages.

Mechanical memory persists when the second seeding stiffness increases, but the number of fates accessible to an MSC decreases, as described in previous sections. In Fig. 5b the second seeding stiffness is 0.9 kPa. When the relative duration of the first seeding is 50 (blue line), MSCs differentiate into osteoblasts according to mechanical memory. When the relative duration of the first seeding is 15 (red line), MSCs differentiate into myocytes (again, influenced by memory). When the relative duration of the first seeding is 5, 0.5 or 0, however (brown, pink or black lines), MSCs differentiate into adipocytes: mechanical memory is not present when the second seeding duration is less than 15.

Figure 5c shows the dynamics of the system when the second seeding stiffness is 12 kPa. For the longest first seeding duration (blue line), MSCs differentiate into osteoblasts, as above, but when the duration is 15 or lower (red, brown, pink or black lines), MSCs differentiate into myocytes. These data illustrate that as the second seeding stiffness increases, the range of first seeding durations over which mechanical memory is observed decreases, which is consistent with the observation from Yang et al [8]. At a second seeding stiffness of 12 kPa, the memory effect is observed only for osteogenic differentiation, and not for any other lineages. Intriguingly, higher first seeding stiffness values for shorter periods of time might accelerate an MSC towards lineage commitment. *TUBB3* expression approaches the steady state quickly following stimulation on a 30 kPa substrate for a relative time of 0.5 (Fig. 5a, pink line). Compare this to the differentiation characteristics of an MSC seeded only on a 0.3 kPa substrate (Fig. 5a, black line); the latter takes a longer time to differentiate.

### Feedback signaling onto the effective substrate adhesion area

**Fig. 6:**
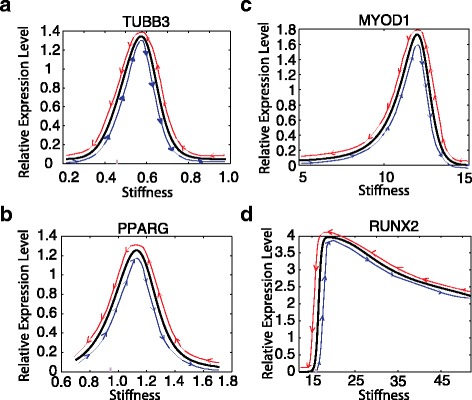
The MSC network precludes multistability when feedback loops are blocked. Shown are the steady states of TUBB3 (**a**), PPARG (**b**), MYOD1 (**c**), and RUNX2 (**d**) under different stiffness values. In each figure the x-axis denotes the stiffness and the y-axis denotes the relative expression levels of specific lineage genes at steady states (*black lines*). The blue lines illustrate how the relative gene expression at the steady state changes as the stiffness increases. The red lines illustrate how the relative gene expression level at the steady state changes as the stiffness decreases

Mechanotransduction pathways may contain positive feedback loops in which integrin engagement activates actomyosin cytoskeleton contractility, resulting in morphological changes affecting the adhesion area of the substrate [1, 17, 18, 23–27]. Here we assess the importance of such feedback. Figure 6 shows the relative expression levels of the lineage-specific genes at steady states for a range of substrate stiffness values. In Fig. 6a, we block the feedback from *TUBB3* onto the effective substrate adhesion area. We see that the bistability that was observed in Fig. 3 is no longer present: no hysteresis effect can be seen when the substrate stiffness is increased or decreased (illustrated by the blue and red lines). Thus, no mechanical memory effect remains for *TUBB3* during MSCs differentiation. Similar results are obtained for *PPARG* (Fig. 6b), *MYOD1* (Fig. 6c) and *RUNX2* (Fig. 6d) when the final seeding stiffness is 0.9 kPa, 10 kPa and 16 kPa, respectively. The mechanical memory of the genes disappeares when the feedback loops are removed. Collectively our simulation results illustrate that the feedback loops downstream of the stiffness of substrates are necessary for the mechanical memory.

### Noise can induce fate switching during MSC differentiation

**Fig. 7:**
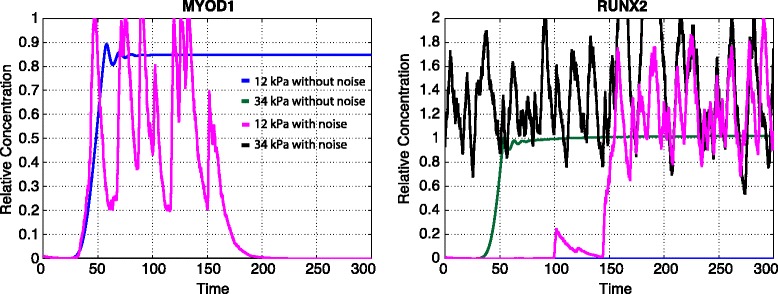
Stochastic gene expression dynamics under different stiffness conditions. The green and blue lines depict the relative expression levels of genes from the deterministic model. The magenta and black lines depict the relative expression levels of genes from the stochastic differential equation model with noise term ~ *N*(0,0.05). Blue and magenta lines represent a first-seeding stiffness of 12 kPa, green and black lines represent a first-seeding stiffness of 34 kPa. The final seeding stiffness is 12 kPa in all cases

There is inherent noise in gene expression dynamics [43, 44]. We employ a stochastic differential equation (SDE) model (described in Methods) to study the effects of gene expression noise on MSC differentiation [45, 46]. We find that SDE simulations broadly recapitulate the results obtained in the deterministic case, however under certain conditions fate switching is observed. In Fig. 7 we simulate a system of SDEs based on the deterministic model with multiplicative noise added to the expression level of each gene; blue and dark green lines describe the relative gene expression under the deterministic model, while pink and black lines describe analogous results under the SDE model. We vary the initial seeding stiffness while keeping the second seeding stiffness constant at 12 kPa. In the deterministic case, we see that *MYOD1* is expressed when the value of the initial stiffness is 12 kPa, and not when the value is 34 kPa. Conversely, *RUNX2* is not expressed at an initial stiffness of 12 kPa, but is expressed when the initial stiffness is 34 kPa: here stem cells are differentiating according to mechanical memory.

In the stochastic case, a different picture emerges. First we note that the memory effect observed for osteogenic differentiation in the deterministic case (driven by *RUNX2* expression) is preserved under the stochastic model (Fig. 7 black line). However, in the stochastic case, at 12 kPa, *MYOD1* is expressed transiently: as its expression declines to zero, *RUNX2* is turned on. Thus noise has induced a fate transition between myogenic and osteogenic lineages. At 34 kPa no such transitions are observed: *RUNX2* is expressed constitutively.

## Discussion

Mesenchymal stem cell fate can be controlled by mechanical dosing [1]. Mechanical memory (past mechanical dosing) also affects stem cell fate, particularly when the initial substrate is stiff [8], it is difficult however to experimentally test the effects of mechanical memory over a wide range of culture conditions. Here we have presented a mathematical model that allows such tests to be performed, producing several striking predictions. We first assessed whether the model is able to recapitulate experimental studies, and find that it does agree with evidence showing MSC differentiation into neurons or adipocytes on softer substrates, and myocytes or osteoblasts on stiffer substrates. We then analyzed model behavior over longer timescales, and found that a mechanical memory region exists for each of these MSC-derived cell lineages, with substantial variation in the memory stiffness range for each cell fate. Previously, a memory region has only been observed during osteogenic differentiation, and even then, only qualitative assessment of its behavior was made. We are able to provide bounds on the substrate stiffness ranges permissive of memory effects for all four lineages.

Upon re-seeding MSCs onto a second substrate, the stem cells differentiate according to mechanical memory under certain conditions. We predict that (in addition to the stiffness of the first substrate) the duration of the first seeding also directly influences stem cell memory. By changing only the duration of the initial seeding we can directly influence cell fate. The number of fates accessible to the MSC can also be controlled by the final seeding stiffness. Landscape analysis demonstrates that, for a constant first seeding stiffness and duration, a higher second seeding stiffness limits the number of MSC fates accessible, and that a sufficiently low final seeding stiffness is permissive of differentiation into all four cell fates. We also found that the feedback loop connecting lineage-specific genes to the effective surface adhesion area is critical for the mechanical memory of MSC differentiation. This might be due to integrin—substrate binding, or morphological changes that occur upon differentiation [1, 3, 7, 17].

As well as their direct relevance for in vitro studies, our model predictions also have important implications for the design of regenerative therapeutics. A major challenge here is lack of precision in cell fate control following transplantation. A better understanding of the relationship between mechanical conditions, culture duration, and stem cell fates is needed. By defining the substrate stiffness limits that regulate MSC fates, this study provides means to design experimental protocols that constrain cells to be confined within fate boundaries, thus avoiding differentiation towards an undesirable fate [47–50]. Mechanical memory could be employed advantageously here, e.g. by preconditioning MSCs via mechanical dosing. An improved understanding of the MSC mechanotransduction pathway will also affect our ability to control multipotency, and should enable us to better culture undifferentiated MSCs in vitro.

In order to study additional effects of the mechanotransduction pathway on stem cell fate, a model that describes a larger regulatory network is needed. Cell-cell interactions have not yet been incorporated into our model, although there is a large body of work detailing the importance of the microenvironment (i.e. the effects of cell-cell interactions and of the niche) on stem cell differentiation [30, 51]. In addition, we have chosen a small set of four lineage-specific genes in order to minimize the size of the model’s parameter space. Clearly a greater number of genes are involved in the regulation of MSC fate; without a description of this larger transcriptional network we will not be able to describe nuances of mechanically-induced MSC fate dynamics. However, we believe that the dynamics – and the attractors corresponding to differentiated cell states observed here constitute core pathway mechanisms that would still underlie cell fate decisions in a larger network.

## Conclusions

In this study we sought to investigate the mechanisms of control exerted via mechanical forces upon mesenchymal stem cells during culture and differentiation. Simulations of the gene expression dynamics under different mechanical dosing conditions have led to several predictions. We found that non-cooperative gene regulation is the most plausible mechanism to describe MSC differentiation and we predict that mechanical memory is a general mechanism affecting all of the MSC-derived lineages in this model. We found that the duration of the initial culture and the substrate stiffness during this initial culture are particularly crucial in determining the MSC fates. In addition, we were able to show that a lower final-seeding substrate stiffness permitted a greater number of MSC fates.

Through careful analysis, the ever-expanding body of high-throughput transcriptomic data will enable the study of ever-more complex gene networks. Both the MSC fate transcriptional network structure and the dynamics of the network need to be inferred from data. Spatial interactions, e.g. arising from niche-mediated effects on MSCs, may necessitate a move towards a suitable model framework such as partial differential equations or cell-based (e.g. Cellular Potts) models. Once a clearer picture emerges, it will be possible to extend our model with the incorporation of relevant new signaling interactions. In doing so, we hope to provide further insight into the complex networks of regulation underpinning mesenchymal stem cell fate.

## Methods

### A dynamical model of mesenchymal stem cell fate

We model a simplified gene regulatory network that underpins MSC fate with ordinary differential equations (ODEs) [31, 32].

1 $$ \begin{array}{l}\frac{d\left[ SAA\right]}{ d t}=\underset{\boxed{10}}{\underbrace{k_1\frac{{\left( S/{K}_1\right)}^{n_1}+{\left(\left[ TUBB3\right]/{K}_2\right)}^{n_2}}{1+{\left( S/{K}_1\right)}^{n_1}+{\left(\left[ TUBB3\right]/{K}_2\right)}^{n_2}}}}+\underset{\boxed{11}}{\underbrace{k_2\frac{{\left( S/{K}_3\right)}^{n_3}+{\left(\left[ PPARG\right]/{K}_4\right)}^{n_4}}{1+{\left( S/{K}_3\right)}^{n_3}+{\left(\left[ PPARG\right]/{K}_4\right)}^{n_4}}}}\\ {}+\underset{\boxed{12}}{\underbrace{k_3\frac{{\left( S/{K}_5\right)}^{n_5}+{\left(\left[ MYOD1\right]/{K}_6\right)}^{n_6}}{1+{\left( S/{K}_5\right)}^{n_5}+{\left(\left[ MYOD1\right]/{K}_6\right)}^{n_6}}}}+\underset{\boxed{13}}{\underbrace{k_4\frac{{\left( S/{K}_7\right)}^{n_7}+{\left(\left[ RUNX2\right]/{K}_8\right)}^{n_8}}{1+{\left( S/{K}_7\right)}^{n_7}+{\left(\left[ RUNX2\right]/{K}_8\right)}^{n_8}}}}-{d}_1\left[ SAA\right]\kern0.5em \end{array} $$

2 $$ \frac{d\left[ YAPTAZ\right]}{ d t}=\underset{\boxed{1}}{\underbrace{k_5\left[ SAA\right]}}-{d}_2\left[ YAPTAZ\right] $$

3 $$ \frac{d\left[ TUBB3\right]}{ d t}=\underset{\boxed{2,3}}{\underbrace{k_6\frac{{\left(\left[ SAA\right]/{K}_9\right)}^{n_9}}{1+{\left(\left[ SAA\right]/{K}_9\right)}^{n_9}+{\left(\left[ YAPTAZ\right]/{K}_{10}\right)}^{n_{10}}}}}-{d}_3\left[ TUBB3\right] $$

4 $$ \frac{d\left[ PPARG\right]}{ d t}=\underset{\boxed{4,5}}{\underbrace{k_7\frac{{\left(\left[ SAA\right]/{K}_{11}\right)}^{n_{11}}}{1+{\left(\left[ SAA\right]/{K}_{11}\right)}^{n_{11}}+{\left(\left[ YAPTAZ\right]/{K}_{12}\right)}^{n_{12}}}}}-{d}_4\left[ PPARG\right] $$

5 $$ \frac{d\left[ MYOD1\right]}{ d t}=\underset{\boxed{6,7}}{\underbrace{k_8\frac{{\left(\left[ YAPTAZ\right]/{K}_{13}\right)}^{n_{13}}}{1+{\left(\left[ SAA\right]/{K}_{14}\right)}^{n_{14}}+{\left(\left[ YAPTAZ\right]/{K}_{13}\right)}^{n_{13}}}}}-{d}_5\left[ MYOD1\right] $$

6 $$ \frac{d\left[ RUNX2\right]}{ d t}=\underset{\boxed{8,9}}{\underbrace{k_9\frac{{\left(\left[ YAPTAZ\right]/{K}_{15}\right)}^{n_{15}}}{1+{\left(\left[ SAA\right]/{K}_{16}\right)}^{n_{16}}+{\left(\left[ YAPTAZ\right]/{K}_{15}\right)}^{n_{15}}}}}-{d}_6\left[ RUNX2\right] $$

Where *S* and [*SAA*], are the relative levels of the stiffness (input to the system) and of the effective stiffness adhesion area, respectively. [*YAPTAZ*], [*TUBB*3], [*PPARG*], [*MYOD*1], and [*RUNX*2] denote the relative concentrations of *YAP/TAZ*, *TUBB3*, *PPARG*, *MYOD1,* and *RUNX2*. Since concentration and time in the model are given in relative units, i.e. are dimensionless, then all parameters in the above equations are also dimensionless. *d*_*i*_ (*i* = 1, 2, …, 6) in Eqs. (1,2,3,4,5 and 6) are the degradation rates of the corresponding genes/factors. The terms denoted by the label (1, 2, …, 9) under the brackets in Eqs. (1,2,3,4,5 and 6) are the active/inhibitive regulations acting on [*SAA*], [*YAPTAZ*], [*TUBB*3], [*PPARG*], [*MYOD*1], and [*RUNX*2], where the numbers in rectangle boxes are consistent with the circled indices shown in Fig. 2 [52]. All values of parameters in Eqs. (1,2,3,4,5 and 6) shown in Table 2 are estimated or approximated according to the behaviours that we sought to describe. Parameters values are fit to qualitative features of the biological system [1, 6, 8, 9, 19] (See Additional file 1). The data required performing full inference of the parameters are as-yet unavailable, however the results of our sensitivity analysis show that the models results do not depend crucially on specific values of parameters of the model.

### Cooperative regulation model

The terms (10, 11, 12, 13) in Eq. (1) are based on the non-cooperative regulations of MSCs stiffness sensing. Meanwhile, we model the regulations as the cooperative one and Eq. (1) is rewritten below [53].

7 $$ \frac{d\left[ SAA\right]}{ d t}=\underset{\boxed{10,11,12,13}}{\underbrace{k_1\frac{{\left( S/{K}_1\right)}^{n_1}+{\left(\left[ TUBB3\right]/{K}_2\right)}^{n_2}+{\left(\left[ PPARG\right]/{K}_3\right)}^{n_3}+{\left(\left[ MYOD1\right]/{K}_4\right)}^{n_4}+{\left(\left[ RUNX2\right]/{K}_5\right)}^{n_5}}{1+{\left( S/{K}_1\right)}^{n_1}+{\left(\left[ TUBB3\right]/{K}_2\right)}^{n_2}+{\left(\left[ PPARG\right]/{K}_3\right)}^{n_3}+{\left(\left[ MYOD1\right]/{K}_4\right)}^{n_4}+{\left(\left[ RUNX2\right]/{K}_5\right)}^{n_5}}}}-{d}_1\left[ SAA\right] $$

Rehfeldt et al showed the “switch-like” nonlinear relationship between S and SAA expanding from 0.5 kPa to much large stiffness (>60 kPa) and *TUBB3*, *PPARG*, *MYOD1*, and *RUNX2* are turned on in their specific ranges of stiffness, which are relatively disjoint [52, 53]. In particular, the stiffness range for the myogenic differentiation is far away from the one for adipogenic differentiation. Based the properties of the system, we can rewrite our model into four different submodels under the corresponding stiffness ranges. They are shown as follows.

8 $$ \frac{d\left[ SAA\right]}{ d t}={k}_1\frac{{\left( S/{K}_1\right)}^{n_1}+{\left(\left[ TUBB3\right]/{K}_2\right)}^{n_2}}{1+{\left( S/{K}_1\right)}^{n_1}+{\left(\left[ TUBB3\right]/{K}_2\right)}^{n_2}}-{d}_1\left[ SAA\right] $$

9 $$ \frac{d\left[ SAA\right]}{ d t}={k}_1\frac{{\left( S/{K}_1\right)}^{n_1}+{\left(\left[ PPARG\right]/{K}_3\right)}^{n_3}}{1+{\left( S/{K}_1\right)}^{n_1}+{\left(\left[ PPARG\right]/{K}_3\right)}^{n_3}}-{d}_1\left[ SAA\right] $$

10 $$ \frac{d\left[ SAA\right]}{ d t}={k}_1\frac{{\left( S/{K}_1\right)}^{n_1}+{\left(\left[ MYOD1\right]/{K}_4\right)}^{n_4}}{1+{\left( S/{K}_1\right)}^{n_1}+{\left(\left[ MYOD1\right]/{K}_4\right)}^{n_4}}-{d}_1\left[ SAA\right] $$

11 $$ \frac{d\left[ SAA\right]}{ d t}={k}_1\frac{{\left( S/{K}_1\right)}^{n_1}+{\left(\left[ RUNX2\right]/{K}_5\right)}^{n_5}}{1+{\left( S/{K}_1\right)}^{n_1}+{\left(\left[ RUNX2\right]/{K}_5\right)}^{n_5}}-{d}_1\left[ SAA\right] $$

The difficulty is to determine the values of *K*_1_. If *K*_1_ is less than 1000, the hill function in Equation (7) is saturated for high stiffness levels (> 10,000) and it means that the models cannot distinguish the myogenic differentiation and osteogenic differentiation since Eqs. (10 and 11) both approach the limit $\frac{d\left[ SAA\right]}{ d t}={k}_1-{d}_1\left[ SAA\right]$. If *K*_1_ is greater than 10,000, then the model cannot describe the system for low stiffness levels (< 1000) with that *TUBB3* and *PPARG* cannot express under the low stiffness levels since Eqs. (8 and 9) will respectively approach the limit:

$$ \frac{d\left[ SAA\right]}{ d t}={k}_1\frac{{\left(\left[ TUBB3\right]/{K}_2\right)}^{n_2}}{1+{\left(\left[ TUBB3\right]/{K}_2\right)}^{n_2}}-{d}_1\left[ SAA\right] $$

$$ \frac{d\left[ SAA\right]}{ d t}={k}_1\frac{{\left(\left[ PPARG\right]/{K}_3\right)}^{n_3}}{1+{\left(\left[ PPARG\right]/{K}_3\right)}^{n_3}}-{d}_1\left[ SAA\right] $$

Thus the cooperative regulation model is unable to accurately describe the MSC differentiation system over the range of stiffness values considered.

### Sensitivity analysis

In order to calculate the sensitivities of the parameters shown in Table 2 with respect to the memory and multistability of the system, we sample 1000 values between 0.2 kPa and 42 kPa; they are taken as the stiffness of the system and they are vectorized as the stiffness vector *S*_*b*_. We then calculate the steady states, *Q*_*b*_^*Upper*^ and *Q*_*b*_^*Lower*^, corresponding to the steady states on the lower bifurcation branch (indicated by blue arrowhead lines in Fig. 3c-f, and to the steady states on the upper bifurcation branch (indicated by red arrowhead lines in Fig. 3c-f) for each of the genes: *TUBB3*, *PPARG*, *MYOD1*, and *RUNX2*, using the parameters in Table 2. In order to calculate the sensitivity of each parameter, we perturbe it 1000 times under the constraint of CV (coefficient of variance) = 0.05, and calculate the steady states *Q*_*P*_^*Upper*^ (with the same initial conditions as *Q*_*b*_^*Upper*^), and *Q*_*p*_^*Lower*^ (with the same initial conditions as *Q*_*b*_^*Lower*^). We perform such comparisons – for each of the four genes – for a total of 41 parameters and 1000 perturbations, thus for the parameter vector *P*_*i*_^*j*^ (*i* = 1, 2, …, 41; *j* = 1, 2, …, 1000), i.e. the *j-*th perturbation of the *i-*th parameter. We count the number (*N*_*i*_) of *P*_*i*_^*j*^ that satisfies ||*Q*_*P*_^*Upper*^ − *Q*_*b*_^*Upper*^||_2_ + ||*Q*_*P*_^*Lower*^ − *Q*_*b*_^*Lower*^||_2_ < *TOL*.The tolerance, *TOL*, is set such the perturbed parameter vector  gave rise to the same number of steady states as for the unperturbed case (i.e. multistability and the memory effect is maintained; we set *TOL* = 4). The robustness *R*_*i*_ of the *i*-th parameter is defined as $\frac{N_i}{10}\%$ and the sensitivity *S*_*i*_ of the i-th parameter is $1-\frac{N_i}{10}\%$. The robustness values for each of the 41 parameters are shown in the bar graph (See Fig. 3g) and the index of the parameters in the graph is consistent with the one in Table 2. Four of them are sensitive than the rest and they are marked by yellow arrows in the following bar graph.

### Steady state analysis

We compute the steady states of the dynamical system under different *S* in Eqs. (1, 2, 3, 4, 5 and 6). Here we use the continuation method to compute the steady states and their branches [54, 55].

### Landscape potential using a mean field self-consistent approximation and Gaussian approximation

Here we derive an approximation for the potential energy of the system. Starting from the Fokker-Planck equation, we calculate the steady state probability distributions using a self-consistent mean field method [56–58]. The probability function *P*(*X*,*t*) satisfies the following diffusion equation:

12 $$ \frac{\partial P\left( X, t\right)}{\partial t}=-\frac{\partial }{\partial X}\left[ F\left( X, S\right) P\left( X, t\right)\right]+ D\frac{\partial^2}{\partial {X}^2}\left[ d(X) P\left( X, t\right)\right] $$

where *F*(*X*,*S*) and *d*(*X*) are the drift and diffusion part respectively and the noise is weak, i.e. *D<< *1. Note that X is a vector of species ([*SAA*],[*YAPTAZ*],[*TUBB3*],[*PPARG*],[*MYOD1*],[*RUNX2*]) but we have dropped the arrow notation for convenience below. We factor the original probability function using the self-consistent mean field approach [59], $P\left( X, t\right)={\displaystyle \prod_{i=1}^n P\left({X}_i, t\right)}$ to reduce the computational complexity of solving the original equation on the probability, similar to a previous study [57]. We use the Gaussian distribution to approximate the true distribution [57], leading to a description for the mean and variance of the gene expression:

13 $$ {\overline{X}}^{\prime }(t)= F\left(\overline{X}(t), S\right) $$

14 $$ {\sigma}^{\prime }(t)=\sigma (t){A}^T(t)+ A(t)\sigma (t)+2 D\left[\overline{X}(t)\right] $$

where $\overline{X}$ is the mean value of *X*(*t*), *σ*(*t*) is the variance matrix, the matrix element *α*_*ij*_ (*t*) of *A*(*t*) is $\frac{\partial {F}_i\left(\overline{X}(t)\right)}{\partial {\overline{X}}_j(t)}$, i.e. A is the Jacobian matrix.

Since we consider the steady states, then we need to compute ${\overline{X}}^{(j)}\left(\infty \right)$ and *σ* ^(*j*)^(∞) from ${\overline{X}}^{\prime }(t)=0$ and *σ*′(*t*) = 0, for *j* = 1,2,…,*m* respectively, where *m* is the number of basins of attraction. We consider only diagonal elements of *σ* ^(*j*)^(∞) from mean field splitting approximation. For each variable ${{\overline{X}}_i}^{(j)}\left(\infty \right)$, the probability distribution can be estimated using the mean and variance and based on Gaussian approximation [57, 60].

15 $$ {P}^{(j)}\left({X}_j,\infty \right)=\frac{1}{\sqrt{2\pi {\sigma}^{(j)}\left(\infty \right)}} \exp \left[-\frac{{\left[{X}_i{\overline{X}}_i^{(j)}\left(\infty \right)\right]}^2}{2{\sigma}^{(j)}\left(\infty \right)}\right] $$

If *m* = 1, we can use Eq. (6) to compute the probability distribution of the single basin of attraction. If *m* > 1, then the system permits multistability, and for each basin of attraction we compute its probability distribution. The probability function thus becomes a weighted sum of the probabilities given for each basin of attraction,

$$ P\left({X}_i,\infty \right)={\displaystyle \sum_{j=1}^m{\omega}_j{P}^{(j)}\left({X}_i,\infty \right)} $$

where *ω*_*j*_ is the weighting coefficient of the *j*-th basin. Assume *m* attractors, then the number of simulations that end up in each attractor is *N*_1_, *N*_2_, …, *N*_*m*_. The weighting coefficient for the *j*-th basin is then calculated as ${\omega}_j={N}_j/{\displaystyle \sum_{i=1}^m{N}_i}$. Finally, we calculate the potential landscapes based on *U*(*X*) = − ln *P*(*X*, ∞) [61, 62].

### A stochastic differential equation model

A stochastic differential equation (SDE) model for the regulatory network can be constructed via the addition of a noise term [43, 45, 46, 63]:

16 $$ d X(t)= F\left( X(t), S\right) d t+\eta X(t) d W(t) $$

where *W*(*t*) denotes the scalar white noise (or Wiener process), and *η* is the noise coefficient.

## Additional file

Additional file 1: Supplementary information. This PDF contains supplementary results, references and Figures S1-S3 that are not included in the main text. (PDF 191 kb)

## Abbreviations

ECM: Extracellular matrix
MSC: Mesenchymal stem cell
MYOD1: Myogenic Differentiation 1
ODE: Ordinary differential equation
PPARG: Peroxisome proliferator-activated receptor gamma
RUNX2: Runt-related transcription factor 2
SAA: Effective stiffness adhesion area
SDE: Stochastic differential equation
TAZ: Transcriptional coactivator with PDZ-binding motif
TCPS: Tissue culture polystyrene
TUBB3: The gene encode Tubulin beta-3 chain
YAP: Yes-associated protein
