# Supplementary material for: A mathematical model of mechanotransduction reveals how mechanical memory regulates mesenchymal stem cell fate decisions
Source: BMC Syst Biol. 2017 May 16;11:55. doi: 10.1186/s12918-017-0429-x (PMC5434622; doi:10.1186/s12918-017-0429-x)
Supplement: Additional file 1: — Supplementary information. This PDF contains supplementary results, references and Figures S1-S3 that are not included in the main text. (PDF 191 kb) [file 12918_2017_429_MOESM1_ESM.pdf]

Additional file for "A Mathematical Model of Mechanotransduction Reveals How Mechanical Memory Regulates Mesenchymal Stem Cell Fate Decisions"

Tao Peng[1*], Linan Liu[2*], Adam L MacLean[1], Chi Wut Wong[2], Weian Zhao[2], Qing Nie[1,+]

[1] Department of Mathematics, Center for Complex Biological Systems, and Center for Mathematical and Computational Biology, University of California, Irvine, Irvine, CA 92697, USA

[2] Department of Pharmaceutical Sciences, Department of Biomedical Engineering, Department of Biological Chemistry, Sue and Bill Gross Stem Cell Research Center, Chao Family Comprehensive Cancer Center & Edwards Life sciences Center for Advanced Cardiovascular Technology, University of California, Irvine, 845 Health Sciences Road, Irvine, CA 92697, USA

* equal-contribution

+ Corresponding author: Qing Nie: qnie@uci.edu

Contents

Figure S1. The trajectory of SAA against the values of stiffness S.

Figure S2. The trajectory of YAP/TAZ against the values of stiffness S.

Figure S3. Comparison of the time point when the marker genes go ON and the time point when the SAA increases significantly.

Regarding the relationship between SAA and S, we observe that the several orders of magnitude of stiffness range, and a hyperbolic relationship, consistent with Figs. 2C and 4B of a previous work Rehfeldt et al [1].

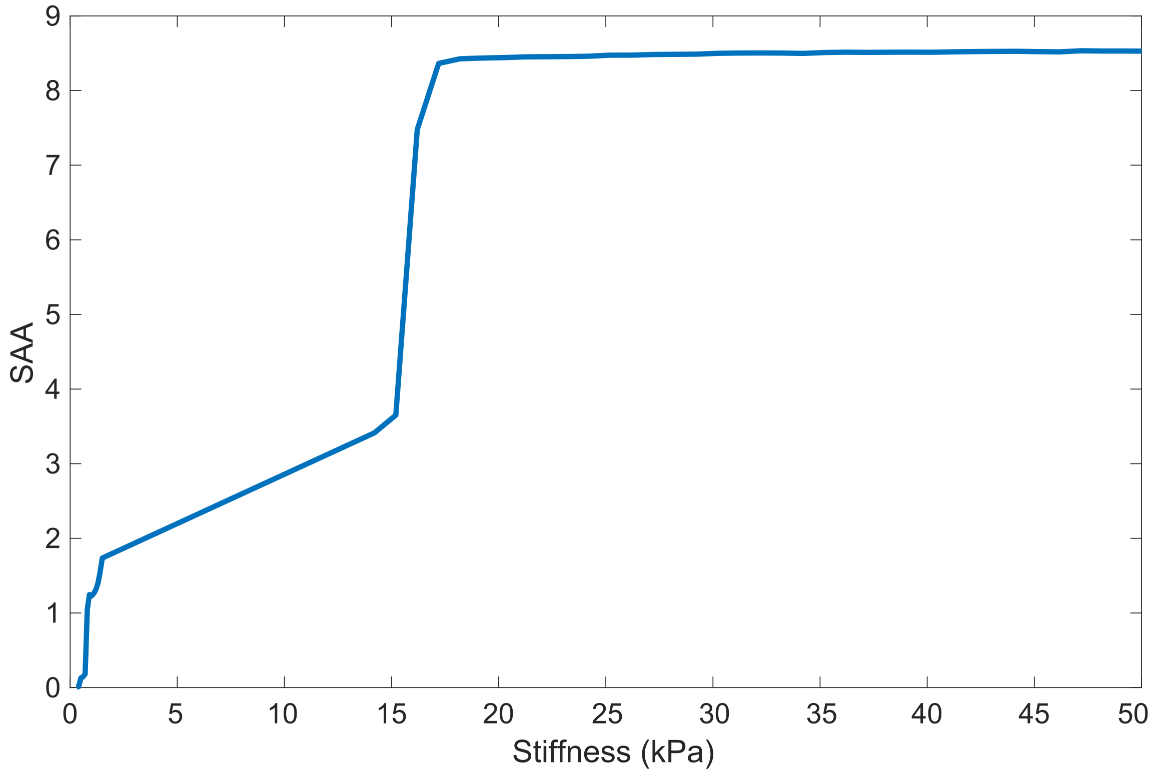

Figure S1. The trajectory of SAA against the values of stiffness S.

In our model, we only implicitly consider the relocalization of YAP/TAZ since the species “YAP/TAZ” in our model is best described as “functional YAP/TAZ”, i.e. the ratio of (nuclear YAP/TAZ: cytoplasmic YAP/TAZ). This is due to several observations: 1) mechano-sensing is tightly coupled to YAP/TAZ relocalization [2,3]; and 2) nuclear YAP/TAZ is the effector form of this species given that we model its ability to modify the transcription of target genes. Below we plot the values of functional YAP/TAZ against the stiffness. The plot demonstrates a more complex relationship that that shown in Swift et al Fig. 4I [3], however again here we note the considerable differences in stiffness scales between the two works.

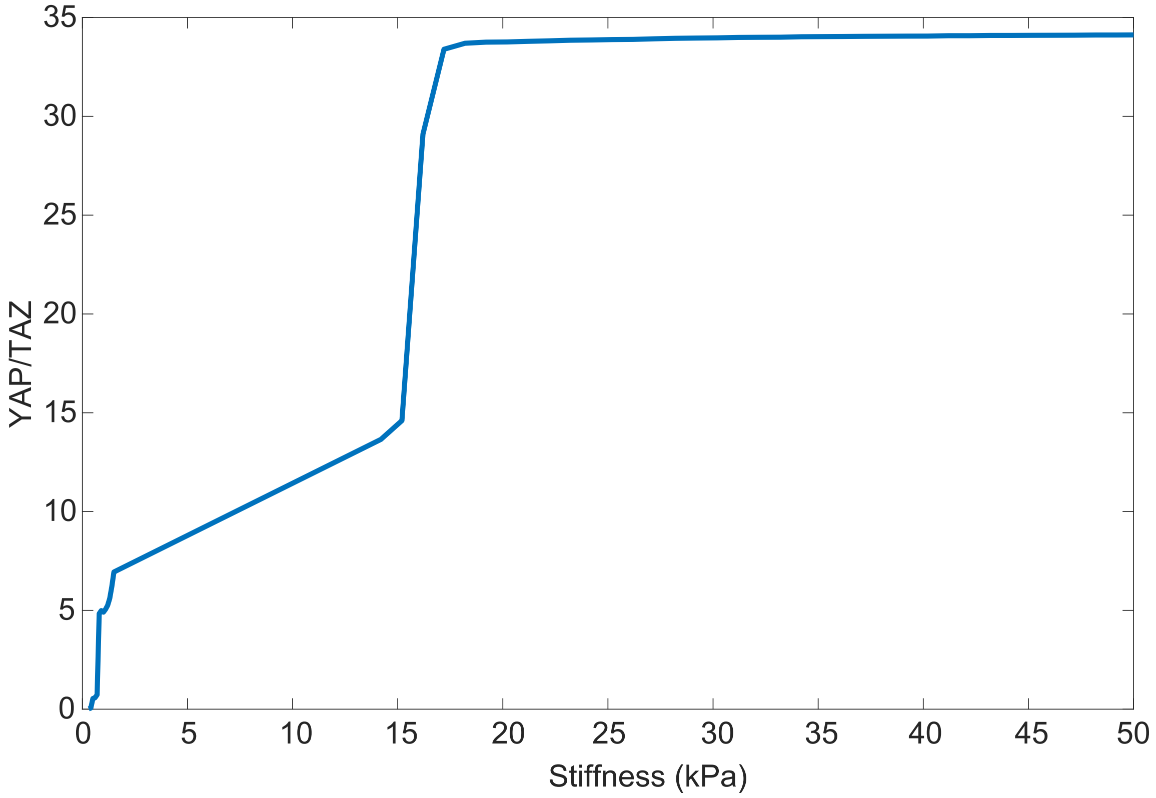

Figure S2. The trajectory of YAP/TAZ against the values of stiffness S.

Below we have plotted the trajectory of SAA overtime under the same conditions as in Figure 5 in the main text. Here we use the single-headed arrows to illustrate when the marker genes go on and the double-headed arrow to illustrate the time point when the SAA increases significantly. As shown, we can see that the double-headed arrows are in each case before the corresponding single headed ones. This shows that SAA increase before differentiation, which is consistent with the observations in the previous experiments [1].

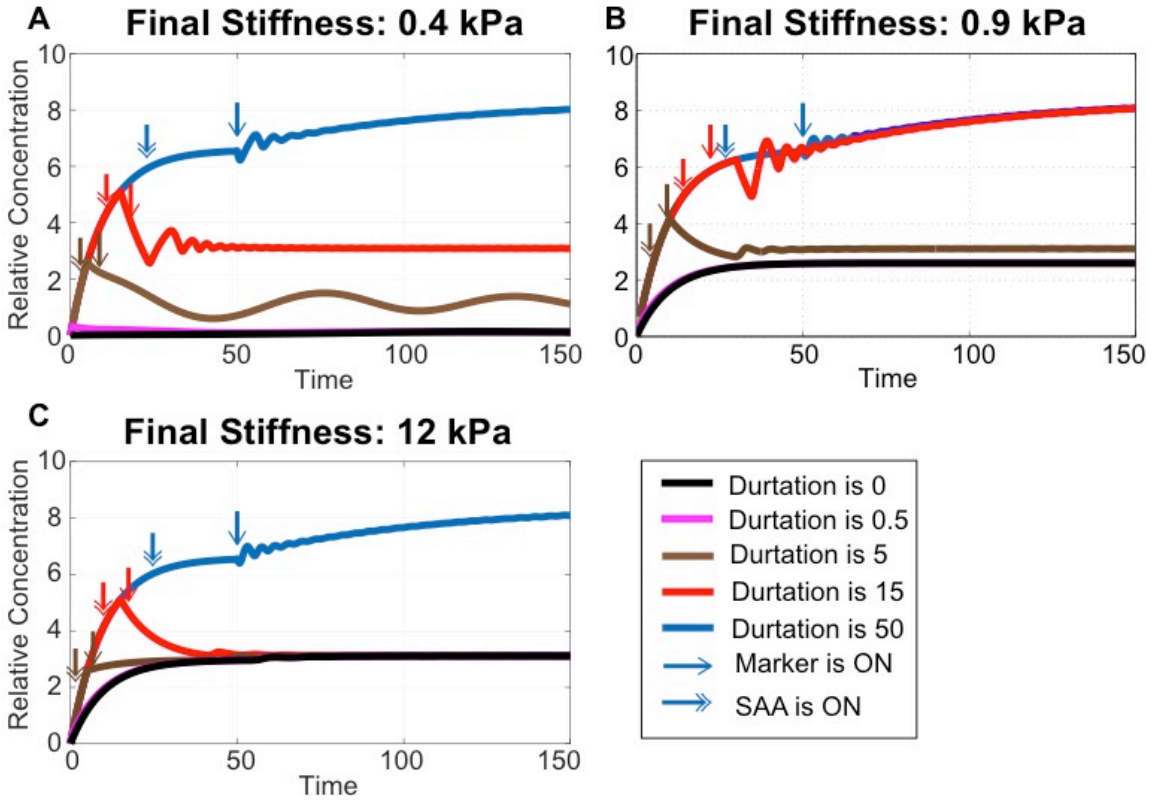

Figure S3. Comparison of the time point when the marker genes go ON and the time point when the SAA increases significantly. The first seeding stiffness in this figure is 30 kPa. The second seeding stiffness is 0.4 kPa (A), 0.9 kPa (B) or 12 kPa (C). Here we use the single-headed arrows to illustrate when the marker genes go on and the double-headed arrow to illustrate the time point when the SAA increases significantly. The different colors are the different durations of the first seeding.

Reference

1. Rehfeldt F, Brown AEX, Raab M, Cai S, Zajac AL, Zemel A, et al. Hyaluronic acid matrices show matrix stiffness in 2D and 3D dictates cytoskeletal order and myosin-II phosphorylation within stem cells. Integrative Biology. 2012;4:422–30.
2. Halder G, Dupont S, Piccolo S. Transduction of mechanical and cytoskeletal cues by YAP and TAZ. Nat. Rev. Mol. Cell Biol. 2012;13:591–600.
3. Swift J, Ivanovska IL, Buxboim A, Harada T, Dingal PCDP, Pinter J, et al. Nuclear lamin-A scales with tissue stiffness and enhances matrix-directed differentiation. Science. 2013;341:1240104–4.
